# The Effect of Depressive and Insomnia Symptoms in Quality of Life among Community-Dwelling Older Adults

**DOI:** 10.3390/ijerph192013704

**Published:** 2022-10-21

**Authors:** Konstantinos Tsaras, Maria Tsiantoula, Dimitrios Papagiannis, Ioanna V. Papathanasiou, Maria Chatzi, Martha Kelesi, Evridiki Kaba, Evangelos C. Fradelos

**Affiliations:** 1 Public Health & Vaccines Laboratory, Faculty of Nursing, School of Health Sciences, University of Thessaly, 41500 Larissa, Greece; 2 Department of Welfare, Municipality of Larissa, 41222 Larissa, Greece; 3 Community Nursing Laboratory, Faculty of Nursing, School of Health Sciences, University of Thessaly, 41500 Larissa, Greece; 4 Department of Infections, University Hospital of Larissa, 41110 Larissa, Greece; 5 Department of Nursing, School of Health and Care Sciences, University of West Attica, 12243 Athens, Greece

**Keywords:** depression, insomnia, quality of life, older adults, community, Greece

## Abstract

The purpose of this study was to investigate the effects of depressive symptoms, insomnia symptoms, and comorbid depressive and insomnia symptoms on the quality of life among community-dwelling older adults in an urban area of central Greece. A cross-sectional study was conducted on 200 older adults (aged ≥ 60) collected from five Open Care Centers for Elderly People of the Municipality of Larissa, Greece. Data were obtained through a questionnaire that included demographic, socioeconomic, and health-related characteristics; the World Health Organization Quality of Life (WHOQoL)-Bref questionnaire; the Geriatric Depression Scale; and the Athens Insomnia Scale. The prevalences of depression, insomnia, and comorbid depression and insomnia were 28% (95% confidence interval (95% CI): 21.8–34.2%), 40.5% (95% CI: 33.7–47.3%), and 19% (95% CI: 13.5–24.5%), respectively. The mean WHOQoL-Bref score for all domains was approximately 14.50, with the highest mean value observed for psychological health (14.79 ± 2.60), followed by the physical health (14.49 ± 2.66), social relationships (14.39 ± 2.03), and environmental domains (14.32 ± 1.90). All WHOQoL-Bref domains were negatively correlated with depression and insomnia. Older adults with depressive symptoms, insomnia symptoms, and comorbid depressive and insomnia symptoms had lower scores in all quality of life dimensions compared with those without.

## 1. Introduction

Life expectancy is increasing across the world, and according to a report by the World Health Organization (WHO), it is estimated that there are over 600 million elderly people, a number that is expected to double by 2025 and reach or exceed 2 billion by 2050 [1]. Several factors can affect longevity, such as heredity, healthy lifestyle, diet, physical exercise, avoiding smoking. Quality of life (QoL) is considered to be related to the way a person perceives his place in life, within the cultural contexts and value system in which he lives, and in relation to his standards, expectations and concerns [2]. The most important factors in the elderly’s quality life are their educational level, income, physical and mental health [3,4].

Older adults face many problems that affect both their physical and mental health. In addition, their functionality is burdened, thus having direct serious effects on their psychological, social, and financial situation. The consequence of all these is a decrease in their self-esteem and self-confidence, feeling as though they are not meeting their social role and social obligations, and ultimately not feeling that they are a “useful” member of society [5]. Organic changes, physical changes, loneliness, and reduced sexual function may be the reasons for their emotional disturbances. These kinds of problems lead to a reduction in their QoL [1].

Depression and insomnia are two of the most frequent disorders among the elderly population [6]. According to a recent systematic review, the prevalence of depression among older adults was estimated to be 28.4%, while in individual studies, depending the screening tool, the percentage has ranged from 15.6% for the DASS-21 to 31.5% for the GDS-15 [7]. Moreover, in another systematic review and metanalysis, the prevalence of depression in older adults was found to be significantly lower among urban (7.5%) than rural (9.6%) residents [8]. In addition, in the context of a large-scale study in Australia and the USA in which 19,114 older adults participated, the prevalence of depressive symptoms was found to be 9.8%, and the female gender, living alone and low educational level were yielded as associated factors [9].

Insomnia is one of the most frequent sleep disorders among older adults. There is evidence that the risk for clinical insomnia steadily increases with age, and it is estimated that 20% of the population aged 65 years and above experience significant and persistent insomnia [10]. In Greece, a large-scale cross-sectional study confirmed the aforementioned age trend in insomnia, as almost 45% of the participants aged >65 years experienced insomnia [11]. Another study found that the prevalence of insomnia among community-dwelling older adults in Greece was 39.2% [6].

Several studies have indicated that both insomnia and depression have severe impacts on the QoL of the elderly population. In a cross-sectional study among community-dwelling elderly Chinese people in which 1168 older adults (aged ≥ 60) participated, the relation between depression and QoL was examined. This study observed negative correlations between physical health, psychological health, environment and depression among older adults [12]. Moreover, a cross-sectional study in Macau in which 451 older adults living in the community and in nursing homes participated revealed that depression, insomnia, and medical conditions could negatively affect QoL in both community-dwelling and institutionalized older adults [13]. Moreover, in another study in China that examined whether depression is a mediator between insomnia and QoL in older adults and the interaction of insomnia and depressive symptoms with QoL among older adults in nursing homes, it was revealed that depressive symptoms could play a mediating role between insomnia and QoL while comorbid insomnia symptoms and depressive symptoms could synergistically interact to affect the health-related QoL [14]. Similar results were extracted by a cross-sectional study in rural areas of China among 871 older adults. The prevalence of symptoms of depression, insomnia, and comorbid depression and insomnia were 34%, 45.7% and 20.3%, respectively, while older adults with symptoms of depression, insomnia, and comorbid depression and insomnia had lower scores in QoL compared with those without [15].

Though the association of depression and insomnia with QoL has been examined, the results have been contradictory [16,17,18,19]. In addition, information about the extent of the role of comorbid depression and insomnia on QoL remains largely unaddressed. Thus, the purpose of this study was to investigate the association between depression, insomnia, and comorbid depression and insomnia on the quality of life in a community-dwelling sample of older adults in the area of Larissa, Greece.

## 2. Materials and Methods

### 2.1. Study Design and Participants

A cross-sectional study was performed. The study population consisted of community-dwelling older adults who were active members and recipients of the services of Open Care Centers for Elderly People (KAPI) of the Municipality of Larissa, whose number amounted to 12,328 people according to the records of the municipality. Larissa is a provincial city located in central Greece, with a population of approximately 200,000. A sample of 200 older adults was collected from the study population. A stratified random sampling procedure per KAPI with gender matching was used when recruiting samples. Specifically, from each one of the five KAPIs included in the study, 40 older adults (20 men and 20 women) were randomly selected. The response rate between KAPIs ranged from 84% to 88%.

The inclusion criteria were as follows: (1) people aged ≥ 60 years, (2) active members of Open Care Centers for Elderly People of the Municipality of Larissa, (3) self-care ability and independent living ability, (4) no history of prior mental disorder or cognitive impairment, and (5) knowledge of the Greek language and ease of communication. The exclusion criteria were (1) individuals with cognitive impairment, (2) individuals with a known history of mental illness, and (3) individuals that refused to provide consent and responses to the questionnaire.

### 2.2. Assessment Instruments

Data were obtained using a self-reported and fully structured questionnaire that consisted of the following four sections.

#### 2.2.1. Health-Related Quality of Life

The 30-item Greek version of the World Health Organization Quality of Life (WHOQoL)-Bref [20] was used to assess the health-related QoL in older individuals. The WHOQoL-Bref questionnaire is a short, 26-item version of WHOQoL-100 developed by the QoL researchers at the WHO as a suitable QoL measurement tool for cross-cultural comparison in both clinical groups and healthy individuals. After testing for the fit of national items within this model, the results indicated four new items with the most satisfactory fit indices that were thus included to form a 30-item version; 28 of these items are divided into four specific domains: “physical”, “psychological”, “social”, and “environmental”. Two individual items assess the perception of overall QoL and general health and constitute the “overall QoL/general health” domain. The items in the WHOQoL-Bref are scored on a five-point Likert scale ranging from 1 (“very dissatisfied”) to 5 (“very satisfied”). Scores in each domain are determined by calculating means from all items contained in a given domain. Mean scores are then multiplied by 4. For each domain, the score ranges from 4 to 20 points. The higher a total score of a domain, the better the individual’s QoL in that specific domain. The 30-item version has well-documented reliability and validity, including high internal consistency, with a Cronbach’s alpha ranging from 0.67 to 0.89 for individual domains. In the present study, the Cronbach’s alpha coefficient per domain ranged from 0.65 to 0.88.

#### 2.2.2. Depressive Symptoms

The Greek version of the Geriatric Depression Scale (GDS-15) [21] was used to identify depressive symptoms in older adults. The GDS short form is a 15-item self-reported psychometric instrument that has been validated to indicate depression symptoms in geriatric populations. Its items require a “yes” or “no” response. Ten items indicate the presence of depression when answered positively (yes = 1), while the other five items are indicative of depression when answered negatively (no = 1). The total score of the scale ranges from 0 to 15 points. A higher total score is indicative of a higher degree of depression. A cutoff point of 6/7 on the GDS-15 has shown optimum receiver operating characteristics for diagnosing depression in older Greek people aged 60 years or above, with a sensitivity of 92% and a specificity of 95%. The scale has shown a high internal consistency and reliability, with a Cronbach’s alpha of 0.94. In the current study, the Cronbach’s alpha coefficient was 0.83.

#### 2.2.3. Insomnia Symptoms

The Athens Insomnia Scale (AIS) is an eight-item self-assessment instrument, originally developed in Greek [22], for quantifying sleep difficulty according to the criteria of the International Classification of Diseases, 10th Revision. The first five items pertain to sleep induction, awakenings during the night, final awakening, total sleep duration, and sleep quality. The last three items are associated with well-being, functioning capacity, and sleepiness during the day. Each item is rated on a four-point scale ranging from 0 (“no problem at all”) to 3 (“very serious problem”). Hence, the overall AIS score ranges from 0 to 24 points, with higher scores reflecting more severe sleep difficulties. A cutoff point of 5/6 on the AIS was used to establish the diagnosis of insomnia, with 93% sensitivity and 85% specificity. In the original version, the internal consistency of the AIS, assessed by Cronbach’s alpha was 0.89. In this study, the Cronbach’s alpha coefficient for the scale was 0.80, indicating sufficient internal consistency.

#### 2.2.4. Covariates

The basic demographic (gender, age, marital status, and number of children), socioeconomic (educational level, employment status, monthly income, and living status), and health-related characteristics (weight, height, and chronic diseases) of the participants were recorded. Marital status was classified as married or unmarried (including divorced and widowed). Body mass index (BMI) was calculated from self-reported height and weight as weight/height^2^ and categorized as follows, according to the WHO classifications: ≤18.4 kg/m^2^ (underweight), 18.5–24.9 kg/m^2^ (normal), 25.0–29.9 kg/m^2^ (overweight), and ≥30 kg/m^2^ (obese).

### 2.3. Data Collection

Data collection was carried out at the five KAPIs of the Municipality of Larissa during the months of September to December 2019 after obtaining permission from the competent municipality office. The questionnaires were completed through face-to-face interviews by the researcher during the stay of the older adults in the KAPIs. Prior to the interview, all participants were given explanations of the purpose of the survey and every participant provided informed consent. The research was conducted following the principles of confidentiality, anonymity, and informed consent outlined by the Declaration of Helsinki and its subsequent revisions.

### 2.4. Data Statistical Analysis

Both descriptive and inferential statistical methods were used to analyze the collected empirical data. Continuous variables are presented as the mean, standard deviation, and range (min–max), while categorical variables are presented as absolute (*n*) and relative (%) frequencies. The scores of the WHOQoL-Bref domains were used as the outcomes (dependent variables), while the occurrence of depression, insomnia, and comorbid depression and insomnia were used as the determinants (independent variables). Pearson correlation analysis was used to explore the relationships between depression (GDS-15 total score), insomnia (AIS total score), and quality of life (WHOQoL-Bref domain scores). An analysis of covariance (ANCOVA) approach was used to determine the extent to which depression, insomnia, and comorbid depression and insomnia could affect the QoL of community-dwelling older adults after the selected variables were controlled from the analysis. The controlled variables were gender, age, marital status, number of children, educational level, employment status, monthly income, living status, body weight status (body mass index), and self-reported chronic diseases. Health-related QoL was separated into physical health, psychological health, social relationships, environmental, and overall QoL/general health. The five endpoints for QoL with respect to depression, insomnia, and comorbid depression and insomnia are reported as marginal means with 95% confidence intervals. All reported *p*-values were two-tailed, and the level of acceptable significance was set to *p* < 0.05. Data analyses were performed using the Statistical Package for Social Sciences (SPSS) software, Version 22.0 (IBM Corp., Armonk, NY, USA).

## 3. Results

### 3.1. Summary of Sociodemographic and Health-Related Characteristics

A total of 200 older adults living in the community participated in this study (100 men and 100 women). The mean age was 75.11 years (SD = 7.89), and the highest percentage of participants was in the age range of 70–79 years (37.5%). Overall, the majority of the respondents were married (56.5%), had one to two children (73%), were primary school graduates or had completed some of its classes (57%), and were not working at present (70.5%). For health-related factors, 47.5% of the older adults were overweight, 22% were obese, and 42.5% reported chronic diseases. The detailed demographic, socioeconomic, and health-related characteristics of the sample are illustrated in Table 1.

### 3.2. Depression, Insomnia, and Comorbid Depression and Insomnia in the Elderly

The screening method of the GDS-15 indicated 144 participants without depression (72%) and 56 with depression (28%; 95% confidence interval (95% CI): 21.8–34.2%). To be exact, 22.5% (95% CI: 16.6–28.4%) of the older adults experienced moderate depression and 5.5% (95% CI: 2.1–8.9%) experienced severe depression. The mean overall GDS-15 score was 4.02 (SD = 3.44; ranging from 0 to 14). According to the screening method of the AIS, the prevalence of insomnia for the total sample was 40.5% (95% CI: 33.7–47.3%). The mean overall AIS score was 5.88 (SD = 4.05; ranging from 0 to 20). The prevalence of comorbid depression and insomnia was 19% (95% CI: 13.5–24.5%) (Table 2).

### 3.3. Quality of Life in the Elderly

Table 3 presents the WHOQoL-Bref domain scores. According to the mean scores of the WHOQoL-Bref dimensions, the highest value was displayed by the psychological health domain (14.79 ± 2.60), followed by the physical health domain (14.49 ± 2.66), and finally the social relationships (14.39 ± 2.03) and environmental (14.32 ± 1.90) domains. The mean score of the overall QoL/general health domain was 14.20 (SD = 2.86; ranging from 8.00 to 20.00). In all WHOQoL-Bref domains, the median score was greater than the midpoint of the measurement scale of the responses, i.e., a value of 12, indicating that the majority of older adults had a relatively high quality of life assessment values and general satisfaction with their health.

### 3.4. Correlation of Depression and Insomnia with Quality of Life in the Elderly

#### 3.4.1. Bivariate Analysis

The relationships between depressive symptoms and health-related QoL and between insomnia symptoms and health-related QoL among community-dwelling older adults were examined using Pearson’s correlation coefficients (Table 4). All WHOQoL-Bref domains were significantly negatively associated with the overall GDS-15 score and the overall AIS score. In particular, when the GDS-15 score increased, older adults’ QoL regarding physical health (r = −0.686), psychological health (r = −0.800), social relationships (r = −0.660), environmental quality of life (r = −0.615), and overall QoL/general health (r = −0.659) decreased. Similarly, when the AIS score increased, older adults’ QoL regarding physical health (r = −0.540), psychological health (r = −0.429), social relationships (r = −0.315), environmental quality of life (r = −0.290), and overall QoL/general health (r = −0.423) decreased.

#### 3.4.2. Multivariate Analysis

The health-related QoL scores between depression and non-depression groups, between insomnia and non-insomnia groups, and between comorbid depression and insomnia and non-comorbid depression and insomnia groups were compared using the ANCOVA approach after controlling for the potentially confounding effects of demographic, socioeconomic, and health-related characteristics (Table 5). The findings showed statistically significant differences in the mean values between the compared groups for all WHOQoL-Bref domains, meaning that depression, insomnia, and comorbid depression and insomnia had significant negative impacts on QoL. Specifically, older adults with depressive symptoms had lower physical health (F = 36.480; *p* < 0.001), psychological health (F = 93.255; *p* < 0.001), social relationships (F = 45.537; *p* < 0.001), environmental quality of life (F = 19.263; *p* < 0.001), and overall QoL/general health (F = 24.696; *p* < 0.001) compared with those without. Participants with insomnia symptoms too had lower physical health (F = 61.618; *p* < 0.001), psychological health (F = 24.662; *p* < 0.001), social relationships (F = 7.589; *p* = 0.006), environmental quality of life (F = 9.335; *p* = 0.003), and overall QoL/general health (F = 17.959; *p* < 0.001) than those without. Finally, older adults with comorbid depression and insomnia also had lower physical health (F = 29.784; *p* < 0.001), psychological health (F = 62.035; *p* < 0.001), social relationships (F = 25.202; *p* < 0.001), environmental quality of life (F = 9.038; *p* = 0.003), and overall QoL/general health (F = 5.464; *p* = 0.020) than those without.

## 4. Discussion

The aim of this study was to examine symptoms of depression and insomnia in older adults and the association of the same with their quality of life. To the best of our knowledge, this was the first study in regional Greece that examined the effect that depression and insomnia can have on the health-related quality of life of older adults.

According to our results, 22.5% of the sample experienced moderate depression, 5.5% experienced severe depression, 40.5% experienced insomnia, and 19% experienced comorbid depression and insomnia. These results are in accordance with previous studies conducted in Greece, in which the prevalence of moderate depression was found to be 22%, that of severe depression was 6.4%, and that of insomnia was 39.2% [6]. In a recent meta-analysis of 42 studies considering 57,486 adults, the average expected prevalence of depression among the elderly was found to be 31.74% [23]. It is widely accepted that depression is a very common disorder in older adults and that the risk of developing depression increases over time. There are various factors that seem to be related to depression, such as socioeconomic, geographic, and health-related factors. Nonetheless, late-life depression is a major public health concern, and regular screening, along with preventive measures, seems to be an effective way to address this problem [7]. Though insomnia was just considered to be one of the symptoms of depression for many years, it has come to be considered a separate disorder and a risk factor for depression in recent years [24]. Despite this change, the specific mechanism and the link between depression and insomnia remain unclear. According to research, various factors can mediate or moderate the link between depression and insomnia, including socio-demographic characteristics (such age, gender, and social support), and a number of neurobiological factors (such as stress) may mediate/moderate the insomnia–depression link [25,26]. The prevalence of comorbid depression and insomnia was found to be 19%, and this finding is consistent with previous studies that indicated a bidirectional relationship between those two conditions [6,15].

The bivariate analysis between depressive symptoms and health-related QoL and between insomnia symptoms and health-related QoL revealed that all of the WHOQoL-Bref domains were significantly negatively associated with the overall GDS-15 score and the overall AIS score. These findings are consistent with previous studies indicating this negative relation, not only in older adults but also in various clinical and non-clinical populations [27]. More specifically, in a cross-sectional study of 323 older adults living in nursing homes in China, the same negative relation was observed [14]. Similar results were further observed within the context of another large-scale study in China, this time among 1168 community-dwelling older adults in which depression was found to be negatively related to the participants’ QoL [12]. Those findings confirm the notion that the QoL of individuals can be affected by various factors, both internal and external. According to the conceptual framework for health-related QoL of Wilson and Cleary, there is a link between clinical variables and health-related QoL. In this case, insomnia and depression are considered to be physiological and psychological factors, respectively, that influence health-related QoL [16,28].

Finally, according to our results, both depression and insomnia have negative effects on health-related QoL among older adults, and those who had comorbid depression and insomnia reported a lower QoL in all domains. These findings are consistent with previous studies examining the effect that depression, insomnia, and comorbid depression and insomnia can have on the QoL of older adults [14,15]. For example, a recent study that sought to evaluate the relationship between sleep quality, duration, and health-related QoL among older adults in the United Kingdom discovered that poor sleep quality and duration were independently linked with worse health-related QoL in the studied population [29]. This could be explained by the fact that poor sleep depletes energy during the day and has a detrimental impact on daily function. Furthermore, a lack of sleep is linked to an increase in negative mood. These consequences have a significant negative impact on health-related QoL [30]. Another study found that even mild depression had an effect on health-related QoL in older adults regardless of socioeconomic status, gender, or age [31].

This study had some inherent limitations. First of all, self-administered psychometric instruments focus on subjective symptoms, so they cannot replace objective clinical criteria. Secondly, the self-reporting of health-related characteristics may reduce the internal validity of research due to information bias (recall and social acceptance). Thirdly, data were exclusively derived from members of Open Care Centers for Elderly People and not from the general elderly population. Thus, the use of a single study site diminishes the generalizability of the study findings. Finally, data were collected in a cross-sectional survey, so the associations among depression, insomnia, and QoL should be interpreted with caution, as a cross-sectional design cannot establish causality. Despite these limitations, this community-based study confirms most findings reported by other authors, is an important accession to the literature, and provides additional evidence on the impact of depressive symptoms, insomnia symptoms, and comorbid depressive and insomnia symptoms in QoL among older adults using stratified random sampling with gender matching and standardized research instruments.

## 5. Conclusions

In conclusion, this study found that symptoms of depression and insomnia were common in older adults living in an urban community in Greece. Moreover, subjects with symptoms of depression, insomnia, and comorbid depression and insomnia had a poorer quality of life than those without. These findings indicate the importance of developing regular screening programs for depression and insomnia, as well as designing targeted intervention services for the elderly population.

## Figures and Tables

**Table 1:** Sociodemographic and health-related characteristics of the participants (*n* = 200).

Characteristics	Categories	*n*	%
Gender	Male	100	50.0
	Female	100	50.0
Age (years)	60–69	60	30.0
	70–79	75	37.5
	≥80	65	32.5
Marital status	Married	113	56.5
	Single	10	5.0
	Divorced	9	4.5
	Widowed	68	34.0
Number of children	0	15	7.5
	1–2	146	73.0
	≥3	39	19.5
Highest level of education	No formal education	38	19.0
	Primary	76	38.0
	Secondary	59	29.5
	Tertiary	27	13.5
Working at the present	Yes	59	29.5
	No	141	70.5
Monthly income (in Euro)	≤500	30	15.0
	501–1000	96	48.0
	˃1000	74	37.0
Living arrangement	Alone	67	33.5
	With family	133	66.5
Body mass index (kg/m^2^)	18.5–24.9 (Normal)	61	30.5
	25.0–29.9 (Overweight)	95	47.5
	≥30.0 (Obese)	44	22.0
Self-reported chronic diseases	Yes	85	42.5
	No	115	57.5

**Table 2:** Prevalence of depression, insomnia, and comorbid depression and insomnia among older adults (*n* = 200).

GDS-15 Score	*n*	%	Mean ± SD	Range
≤6 (Absence of Depression)	144	72.0		
7–10 (Moderate Depression)	45	22.5		
11–15 (Severe Depression)	11	5.5		
Overall Scale			4.02 ± 3.44	0–14
**AIS Score**	***n***	**%**	**Mean ± SD**	**Range**
≤5 (Absence of Insomnia)	119	59.5		
≥6 (Presence of Insomnia)	81	40.5		
Overall Scale			5.88 ± 4.05	0–20
**Comorbid Depression and Insomnia**	***n***	**%**		
No	162	81.0		
Yes	38	19.0		

GDS: Geriatric Depression Scale; AIS: Athens Insomnia Scale; SD: standard deviation.

**Table 3:** Scores of the WHOQoL-Bref (30-item Greek version) dimensions among older adults (*n* = 200).

WHOQoL-Bref Domains	Item Amount	Mean ± SD	Median	Range
Physical Health	9	14.49 ± 2.66	14.67	7.56–20.00
Psychological Health	6	14.79 ± 2.60	15.33	6.67–20.00
Social Relationships	5	14.39 ± 2.03	14.40	6.40–18.40
Environment	8	14.32 ± 1.90	14.50	8.00–19.50
Overall QoL/General Health	2	14.20 ± 2.86	14.00	8.00–20.00

Note: Potential score for each WHOQoL-Bref domain ranges from 4 to 20. WHOQoL: World Health Organization Quality of Life; SD: standard deviation.

**Table 4:** Pearson correlations of GDS-15 and AIS with WHOQoL-Bref dimensions.

WHOQoL-Bref Domains	GDS-15 Score	AIS Score
Physical Health	−0.686 *	−0.540 *
Psychological Health	−0.800 *	−0.429 *
Social Relationships	−0.660 *	−0.315 *
Environment	−0.615 *	−0.290 *
Overall QoL/General Health	−0.659 *	−0.423 *

WHOQoL: World Health Organization Quality of Life; GDS: Geriatric Depression Scale; AIS: Athens Insomnia Scale. * *p* < 0.001.

**Table 5:** ANCOVA test results of depression, insomnia, and comorbid depression and insomnia with WHOQoL-Bref dimensions.

WHOQoL-Bref Domains	Factors	Marginal Means (95% CI)	*F*	*p*-Value	*η* ^2^
Physical Health	Depression		36.480	<0.001	0.163
	No	15.04 (14.74–15.34)			
	Yes	13.07 (12.54–13.59)			
	Insomnia		61.618	<0.001	0.247
	No	15.35 (15.04–15.67)			
	Yes	13.22 (12.83–13.61)			
	Comorbid Depression and Insomnia		29.784	<0.001	0.137
	No	14.87 (14.59–15.15)			
	Yes	12.87 (12.24–13.51)			
Psychological Health	Depression		93.255	<0.001	0.332
	No	15.66 (15.37–15.96)			
	Yes	12.53 (12.01–13.05)			
	Insomnia		24.662	<0.001	0.116
	No	15.44 (15.07–15.82)			
	Yes	13.82 (13.35–14.29)			
	Comorbid Depression and Insomnia		62.035	<0.001	0.248
	No	15.35 (15.06–15.64)			
	Yes	12.37 (11.72–13.03)			
Social Relationships	Depression		45.537	<0.001	0.195
	No	14.95 (14.68–15.22)			
	Yes	12.95 (12.48–13.43)			
	Insomnia		7.589	0.006	0.039
	No	14.71 (14.38–15.04)			
	Yes	13.93 (13.52–14.34)			
	Comorbid Depression and Insomnia		25.202	<0.001	0.118
	No	14.72 (14.46–14.98)			
	Yes	13.00 (12.41–13.59)			
Environment	Depression		19.263	<0.001	0.093
	No	14.67 (14.41–14.93)			
	Yes	13.41 (12.95–13.87)			
	Insomnia		9.335	0.003	0.047
	No	14.64 (14.34–14.93)			
	Yes	13.85 (13.48–14.22)			
	Comorbid Depression and Insomnia		9.038	0.003	0.046
	No	14.50 (14.26–14.75)			
	Yes	13.53 (12.97–14.09)			
Overall QoL/General Health	Depression		24.696	<0.001	0.116
	No	14.74 (14.39–15.10)			
	Yes	12.80 (12.18–13.43)			
	Insomnia		17.959	<0.001	0.087
	No	14.80 (14.39–15.20)			
	Yes	13.33 (12.83–13.83)			
	Comorbid Depression and Insomnia		5.464	0.020	0.028
	No	14.40 (14.06–14.75)			
	Yes	13.35 (12.57–14.13)			

Note: WHOQoL-Bref domains as dependent variables; depression, insomnia, and comorbid depression and insomnia as independent variables; gender, age, marital status, number of children, educational level, employment status, monthly income, living status, body weight status (body mass index), and self-reported chronic diseases as covariates. WHOQoL: World Health Organization Quality of Life; CI: confidence interval.

## Data Availability

Data supporting the findings of this study are available from the corresponding author upon reasonable request.
